# Vascular Endothelium-Dependent and Independent Actions of Oleanolic Acid and Its Synthetic Oleanane Derivatives as Possible Mechanisms for Hypotensive Effects

**DOI:** 10.1371/journal.pone.0147395

**Published:** 2016-01-22

**Authors:** Hlengiwe P. Madlala, Thomas Metzinger, Fanie R. van Heerden, Cephas T. Musabayane, Kanigula Mubagwa, Chantal Dessy

**Affiliations:** 1 School of Laboratory Medicine & Medical Sciences, College of Health Sciences, University of KwaZulu-Natal, Durban, South Africa; 2 Pole of Pharmacology and Therapeutics, Institute of Experimental and Clinical Research, Université catholique de Louvain, Brussels, Belgium; 3 School of Chemistry & Physics, College of Agriculture, Engineering & Science, University of KwaZulu-Natal, Pietermaritzburg, South Africa; 4 Department of Cardiovascular Sciences, Katholieke Universiteit Leuven, Leuven, Belgium; IDIBAPS—Hospital Clinic de Barcelona, SPAIN

## Abstract

**Purpose:**

Plant-derived oleanolic acid (OA) and its related synthetic derivatives (Br-OA and Me-OA) possess antihypertensive effects in experimental animals. The present study investigated possible underlying mechanisms in rat isolated single ventricular myocytes and in vascular smooth muscles superfused at 37°C.

**Methods:**

Cell shortening was assessed at 1 Hz using a video-based edge-detection system and the L-type Ca^2+^ current (I_CaL_) was measured using the whole-cell patch-clamp technique in single ventricular myocytes. Isometric tension was measured using force transducer in isolated aortic rings and in mesenteric arteries. Vascular effects were measured in endothelium-intact and denuded vessels in the presence of various enzyme or channel inhibitors.

**Results:**

OA and its derivatives increased cell shortening in cardiomyocytes isolated from normotensive rats but had no effect in those isolated from hypertensive animals. These triterpenes also caused relaxation in aortic rings and in mesenteric arteries pre-contracted with either phenylephrine or KCl-enriched solution. The relaxation was only partially inhibited by endothelium denudation, and also partly inhibited by the cyclooxygenase (COX) inhibitor indomethacin, with no additional inhibitory effect of the NO synthase inhibitor, N-ω-Nitro-L-arginine. A combination of both ATP-dependent channel inhibition by glibenclaminde and voltage-dependent K^+^ channel inhibition by 4-aminopyridine was necessary to fully inhibit the relaxation.

**Conclusion:**

These data indicate that the effects of OA and its derivatives are mediated via both endothelium-dependent and independent mechanisms suggesting the involvement of COX in the endothelium-dependent effects and of vascular muscle K^+^ channels in the endothelium-independent effects. Finally, our results support the view that the antihypertensive action of OA and its derivatives is due to a decrease of vascular resistance with no negative inotropic effect on the heart.

## Introduction

Hypertension is a highly prevalent cardiovascular condition and constitutes a major risk factor for other cardiovascular diseases [1]. Cardiovascular-related mortality and morbidity pose a heavy burden on health systems, especially in conditions of limited resources in low and middle-income countries [2]. Although there are currently many effective anti-hypertensive drugs, these remain relatively inaccessible to the poor communities due to high cost, especially since the majority of the patients require more than one therapeutic drug. There is, therefore, a need to find cheaper therapeutic alternatives. Plant-derived extracts have been used as therapeutic agents for hundreds of years by many cultures because of their accessibility [3–5]. However, research is needed to validate the efficacy and the action mechanisms of these plant extracts, including those with beneficial effects on hypertension. There is great interest in plant derived triterpenes, including oleanolic acid (OA), which possess multiple beneficial systemic (antiinflammatory, immunomodulatory, antitumor, antidegenerative, antidiabetic) or organ-specific (renoprotective, cardioprotective, hepatoprotective) effects (for references see [6]). We have previously demonstrated that *Syzygium aromaticum*-derived oleanolic acid (OA) and related triterpene derivatives possess antihypertensive effects in experimental models of hypertensive animals [7, 8]. In particular, we have shown that these triterpenes have marked natriuretic and antioxidant actions, particularly in Dahl-salt sensitive (DSS) and spontaneously hypertensive rats (SHR) [8]. The natriuretic action was not accompanied by an effect on diuresis, excluding any effect on intravascular filling. Hence, the interest of this study was to further investigate the mechanisms of blood pressure-lowering properties of OA and related oleanane derivatives, and more specifically to examine potential cardiac and vascular effects.

Vascular smooth muscle contraction and relaxation result from the increased phosphorylation and dephosphorylation, respectively, of myosin light chains. Increased phosphorylation is mediated by the increased activity of a calmodulin-dependent myosin light chain kinase (MLCK) and by the decreased activity of phosphatases (PP). This occurs following the increase of the cytosolic Ca^2+^ concentration ([Ca^2+^]_c_) either upon opening of sarcolemmal Ca^2+^ channels by membrane depolarization or upon Ca^2+^release from intracellular stores following the formation of inositol-triphosphate (IP_3_) by agonists coupled to phospholipase C. Hyperpolarization following the opening of various types of K^+^ channels causes relaxation by deactivating the Ca^2+^ channels, and the role of K^+^ channels in relaxation can be tested using K^+^ channels blockers. MLCK or PP enzyme activities and hence smooth muscle contraction can also be modulated by various signaling pathways, including those involving intracellular nucleotides (such increases of intracellular cGMP or cAMP, mediated by NO and by cyclooxygenase (COX) products, respectively) but also nucleotide-independent cascades. The role of these pathways can be tested by inhibiting key enzymes involved in the signaling cascade.

The aim of our study was to examine the possible role of cardiac negative inotropic and/or vasculo-relaxant effects of OA and derivatives as mechanisms underlying the blood pressure-lowering properties of these agents. In particular, we wanted to examine the pathways implicated in these effects. Therefore we tested the role of ATP-dependent potassium channels in OA and derivative-induced relaxation using glibenclamide, and that of voltage-dependent channels using 4-amino-pyridine. NO formation was blocked by a non-specific NO synthase (NOS) inhibitor, and COX was inhibited by indomethacin.

## Materials and Methods

### Chemicals

OA (C_30_H_47_O_3_) was isolated from *Syzygium aromaticum* [(Linnaeus) Merrill & Perry] (Myrtaceae) (cloves) and its methyl ester (Me-OA; C_31_H_50_O_3_) and brominated (Br-OA; C_31_H_43_BrO_4_) derivatives were synthesized as described previously [8]. Stock solutions of these drugs were prepared in DMSO and were diluted to the final experimental concentration (0.1 μM-1 mM) by dissolving in the cell or tissue superfusing solutions (see below).

### Animals

Male Wistar and Dahl salt-sensitive (DSS) rats (250–300 g) were purchased from Charles River Laboratories Inc. (Wilmington, MA, United States of America) and housed in the Animalium of the University of Leuven. The rats were maintained on a 12 h light / dark regime and had free access to both food and water. The DSS rats were fed high salt Na^+^ diet (8%) (Bio Services, Berlin, Germany) from the age of 4 up to 10 weeks. All experimental protocols were reviewed and approved by animal ethics committees of KULeuven (4500768204) and UCL (2012/MD/UCL/004).

### Cardiac cell and vascular tissue isolation

#### Ventricular cardiomyocyte isolation

The methods for cell dissociation and electrophysiological measurements were similar to those described previously [9]. Briefly, rats were injected with heparin (70–85 mg kg^-1^ i.p) 10 min before sacrifice, and they were euthanized by injection with pentobarbital (150–300 mg kg^-1^ i.p). The chest was cut open and the heart immediately removed and placed in ice-cold cardioplegic solution containing 27 mM KCl and 50 mM glucose to arrest contraction and protect against metabolic consequences of hypoxia during cannulation of the aorta. The heart was then mounted on the perfusion system and was initially perfused with normal Tyrode solution of the following composition in mM: 135 NaCl, 5.4 KCl, 1.8 CaCl_2_, 0.9 MgCl_2_, 10 HEPES, with pH 7.45 to test its functional state and wash out the remaining blood. Cell dissociation involved 1) perfusion with Ca^2+^-free Tyrode for 5–10 min, 2) perfusion with Ca^2+^-free Tyrode containing collagenase A (0.4–0.5 mg mL^-1^, Roche Diagnostics, Germany) and protease (type XIV 0.08 mg mL^-1^, Sigma, USA) to digest the extracellular matrix, 3) perfusion with Ca^2+^-free Tyrode containing collagenase A (without added protease), 4) perfusion with Ca^2+^-and enzyme-free solution to stop the enzyme digestion, and 5) perfusion with Tyrode solution into which Ca^2+^ was gradually reintroduced up to a concentration of 0.18 mM. The ventricles were cut out into the latter solution and cells were dissociated by gentle mechanical agitation and filtered through a mesh (200 μm hole diameter). Cells were given time to settle down at room temperature before use for measurements.

#### Vascular tissue isolation

The methods for vascular tissue isolation, contraction and relaxation measurements were similar to those described previously [10]. Briefly, male Wistar and DSS rats (250 g) were sacrificed by stunning and exsanguination. Aorta or small, 2^nd^ or 3^rd^ branches of mesenteric arteries (SMA) were quickly removed and immersed in physiological saline solution (PSS) of the following composition in mM: 120 NaCl, 5.9 KCl, 25 NaHCO_3_, 11.5 glucose, 1.2 NaH_2_PO_4_, 1.2 MgCl_2_ and 2.5 CaCl_2_. Arteries were cleaned of all fat and connective tissue. All dissection procedures were carefully done to protect the functional endothelium from damage. Each aortic or mesenteric segment was cut with a sterile dissection scissor into rings of approximately 1.5–2 mm width. Endothelium denuded aortic rings and SMA were obtained by gently rubbing the lumen of the artery with forceps tip or with hair, respectively.

### Measurements

#### Cell shortening in cardiac myocytes

Cells were placed in a chamber mounted on the stage of an inverted microscope (Olympus X-70) and superfused with Tyrode solution at 35°C. The cells were field-stimulated with 2.5 ms pulses at a frequency of 1 Hz (Myopacer Cell Stimulator, IonOptix, Milton, USA). A video-based edge detector (Crescent, Salt Lake City, USA) was used to capture and convert changes in cell length during contraction and relaxation into an analog voltage signal. Tyrode solution containing OA or derivatives was superfused at different time intervals during the experimental period. The magnitude of cell contraction was assessed by measuring the amplitude of cell shortening, the kinetics of contraction development by measuring the time-to-peak contraction (TTP) and the kinetics of relaxation by measuring the time constant (tau) of the late, exponential relengthening. Cell contraction is expressed as percentage shortening relative to the resting cell length.

#### Ca^2+^ current measurements

In a few cells, preliminary studies were carried out to examine whether the inotropic effects of the drugs could be accounted for by an effect on the L-type Ca^2+^ current (I_CaL_). For this purpose, whole cell currents were recorded with the patch-clamp technique at room temperature using patch pipettes pulled from capillary glass (GB 200-8P; Science Products, Hofheim, Germany) on a DMZ Universal Puller (Zeitz-Instrumente, Munich, Germany), with a final resistance of 2–4 MΩ when filled with internal solution of the following composition in mM: 130 CsCl, 25 TEA-Cl, 1 MgCl_2_, 5 Na_2_ATP, 0.1 Na_2_GTP, 1 EGTA, 5 HEPES, pH adjusted to 7.25 with CsOH. The voltage-clamp protocol consisted of a pre-step from the holding potential of -70 mV to -50 mV to inactivate voltage-dependent Na^+^ channels, followed by 200 ms depolarizing voltage steps to various levels between -40 and +70 mV to activate I_Ca_. In these experiments, the cells were superfused and internally dialyzed with Cs-based solutions in order to block K^+^ currents [9].

#### Isometric tension and vasodilatory effects

Aortic rings were mounted under a tension of 20 mN in a 12.5 mL organ-bath. Mesenteric arteries were mounted in a wire myograph (DMT, Aarhus, Denmark) under the tension determined by the normalization procedure [11] in a 6 mL organ bath. The baths were perfused with PSS and gassed with 95% O_2_ and 5% CO_2_ at 37°C. Artery rings were first stimulated with low-NaCl high-KCl PSS of the following composition in mM: 31.9 NaCl, 94 KCl, 25 NaHCO_3_, 11.5 glucose, 1.2 NaH_2_PO_4_, 1.2 MgCl_2_ and 2.5 CaCl_2_ in order to assess vessel integrity. A washout and recovery period of 15 min was allowed before starting the experiment.

Aortic rings and SMA with and without functional endothelium were pre-contracted with a single sub-maximal concentration of KCl (50 mM) or phenylephrine (PE; 5 μM). When a sustained and stable contraction was observed, cumulative concentration-response effects were measured upon sequential exposure to carbachol (CCh; 0.01–100 μM), to test the presence of a functional endothelium, or to OA and derivatives (0.1 μM-1 mM). Endothelium-denuded vessels were recognized by the absence of a significant response to the addition of CCh (residual contraction larger than 90% of the maximum contraction). Each drug concentration was left in contact with the vessels for 2 min. The contractile tension was measured using data acquisition hardware and software (Myodacq 2.01, DMT, Aarhus, Denmark). Following each concentration-response curve the vessels were washed with PSS and let to recover in PSS for 15 min. The involvement of NO and/or COX in triterpene-induced effects was examined in intact aortic rings and SMA pre-treated with N-ω-Nitro-L-arginine (L-NoARg; 100 μM) a NOS inhibitor, and/or with indomethacin (INDO; 10 μM), a non-selective COX inhibitor for 30 minutes. To assess a possible involvement of K^+^ channels in the effects of OA and derivatives, isolated vessels were pretreated with the ATP-dependent K^+^ channel blocker glibenclamide (Gli, 5 mM) or/and with the voltage-activated K^+^ channel blocker 4-aminopyridine (4-AP; 1 mM). The drug concentrations used cause maximal blockade of the targeted channels.

### Data analysis

Data analysis for cardiac studies was performed using Clampfit 8.2 (Axon Instruments, Scotland) and Origin (Origin lab, USA). Differences between groups were analyzed for statistical significance using ANOVA with a Fisher post-hoc test, using Statistica 7.1 (StatSoft, USA). Vascular studies data were analyzed using GraphPad InStat (version 5.00, GraphPad Software, San Diego, CA, USA). Statistical comparison of the differences between the control and experimental groups was performed with a two-way analysis of variance, followed by a Bonferroni multiple comparison test. All data are expressed as mean ± standard error of the mean (SEM). When comparing data, a difference with p < 0.05 was considered as significant.

## Results

### Effects on cardiomyocyte shortening

**Fig 1:**
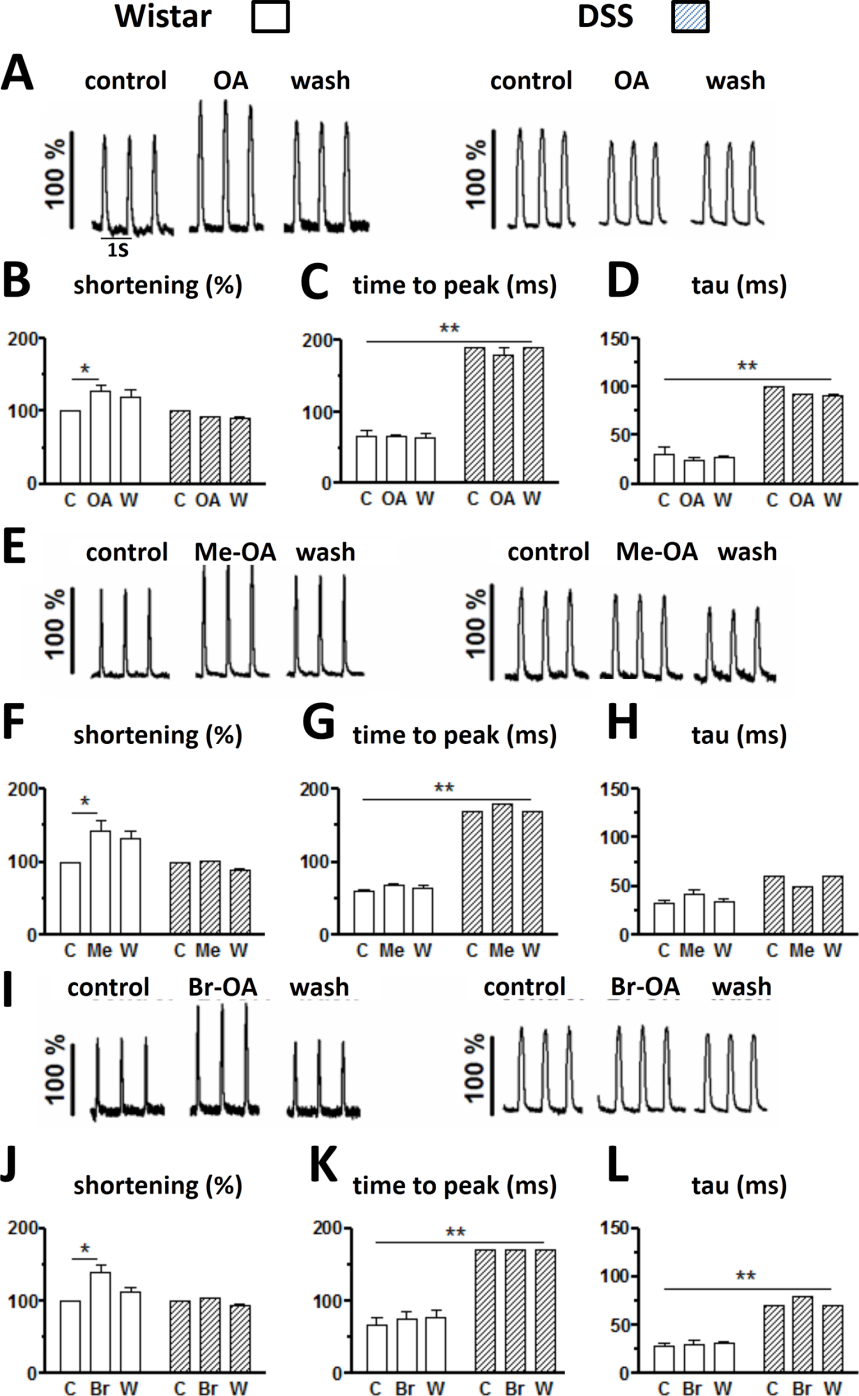
Cell contraction measured under control conditions, during drug treatment and during drug washout in ventricular myocytes from Wistar and DSS rats. Cells were superfused with Tyrode solution containing OA, Me-OA and Br-OA (160 μmol L^-1^) during the treatment period. Original tracings of cell shortening from Wistar (left panels) and DSS rats (right panels) are displayed in A (for OA), E (for Me-OA) and I (for Br-OA). Amplitudes of cell shortening (B, F and J), time to peak shortening (C, G and K) and time constant of relaxation (tau; D, H and L) were measured from Wistar (unfilled columns) and DSS rats (stripped columns) at 1 Hz stimulation frequency (n = 6) for each drug. C: control, OA: oleanolic acid, Me: methylated derivative, Br: brominated derivative, W: washout. * p < 0.001 for drug vs control (short horizontal bars), ** p < 0.0001 for Wistar vs DSS (long horizontal bars).

Fig 1 illustrates the effects of OA, Br-OA and Me-OA (each at 160 μM) on cell shortening measured at 1 Hz in cardiomyocytes from control, non-hypertensive Wistar rats and from DSS hypertensive rats. Superfusion with Tyrode containing OA, Br-OA or Me-OA increased the shortening amplitude of cells isolated from Wistar rats (Fig 1, tracings in left panels). The effects were reversible, at least in part, during the drug washout period. OA and derivatives had no significant effect on cell shortening in cells isolated from DSS rats (Fig 1, tracings in right panels).

Pooled summary data are presented in the lower parts of each panel of Fig 1. Beside peak amplitudes (left column graph of each panel), kinetic parameters, namely the time-to-peak (middle column graph of each panel) and the time constant of the late relaxation phase (right column graph of each panel), were also measured. It is to be noted that there were marked differences in these kinetic parameters between ventricular myocytes from Wistar rats and those from DSS rats. Time to peak (TTP) and relaxation time constants were significantly (p < 0.0001) increased in cells from the hypertensive animals compared to the cells from normotensive Wistar rats. OA and its derivatives had no effects on these kinetics parameters, even in cells from Wistar rats where contraction amplitude increased.

A few preliminary experiments were conducted to test whether an increase in Ca^2+^ currents can account for the positive inotropic effect observed in Wistar rats. In ventricular myocytes (n = 6), OA had no significant effects on the Ca^2+^ current measured using the whole-cell patch-clamp technique (at 0 mV: control 591.0 ±36.55 pA vs OA 556.4 ±32.60 pA).

Because of the above findings further work focused on vascular effects.

### Effects on vascular smooth muscles

The potential vasodilating properties of OA and derivatives was evaluated in rat aortic rings (mounted in classical organ baths) and in 2^nd^ to 3^rd^ order mesenteric arteries (mounted in wire-myographs). Experiments were performed in endothelium-intact and denuded vessels to discriminate between endothelium-dependent and -independent mechanisms, or in the presence of various inhibitors to determine the signaling pathways or effectors involved.

#### Comparison of the vasodilating effects of OA and derivatives

**Fig 2:**
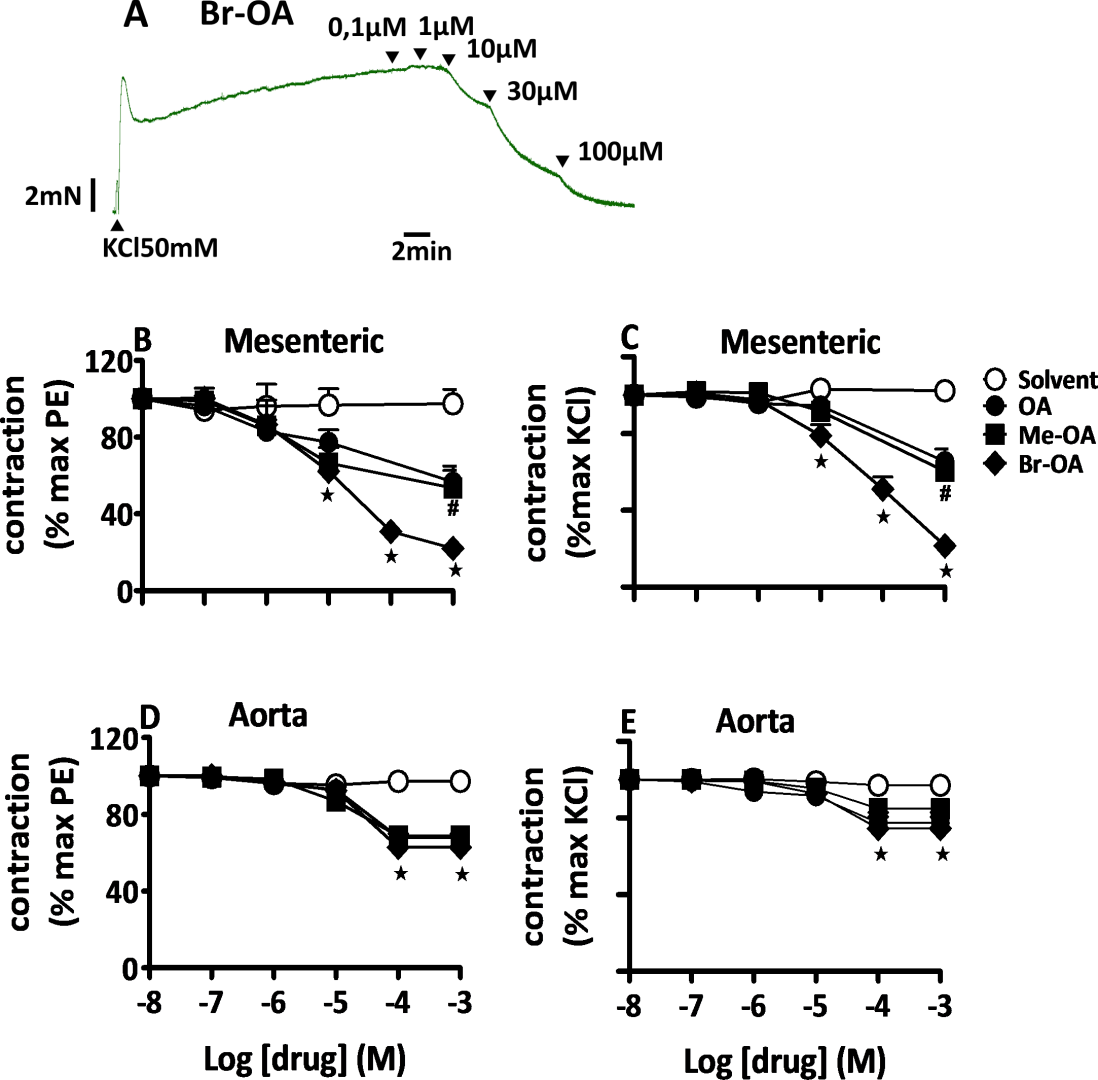
OA and derivatives evoke a vasodilation in aorta and mesenteric arteries from Wistar rats. Original tracing [A] for Br-OA in mesenteric arteries precontracted with KCl. Concentration-response curves for solvent, OA, Me-OA, and Br-OA in endothelium-intact mesenteric arteries [B, C] or aortic rings [C, D] isolated from Wistar rats, pre-contracted with sub-maximal concentration of PE (5 μM) [A, C] and KCl (50 mM) [B, D]. The values shown are means ± SEM (n = 7). * p ˂ 0.001 vs control, **#** vs Br-OA.

Addition of OA, Me-OA or Br-OA on endothelium-intact mesenteric arteries pre-contracted with KCl-enriched solution (Fig 2A and 2C) or PE (Fig 2B) evoked a significant relaxation of the arterial segments isolated from Wistar rats. Similarly, these drugs also induced relaxation in aortic rings (Fig 2D and 2E), but the effects were clearly less marked than in the mesenteric arteries (e.g. p < 0.001, for the maximum relaxation evoked by Br-OA in PE pre-contracted mesenteric vessels vs aorta vessels; p < 0.001 for KCl pre-contracted arteries). When comparing the effects of the three drugs, Br-OA showed a significantly larger maximum relaxation than OA and Me-OA. This difference, however, was not apparent in the aortic rings.

**Fig 3:**
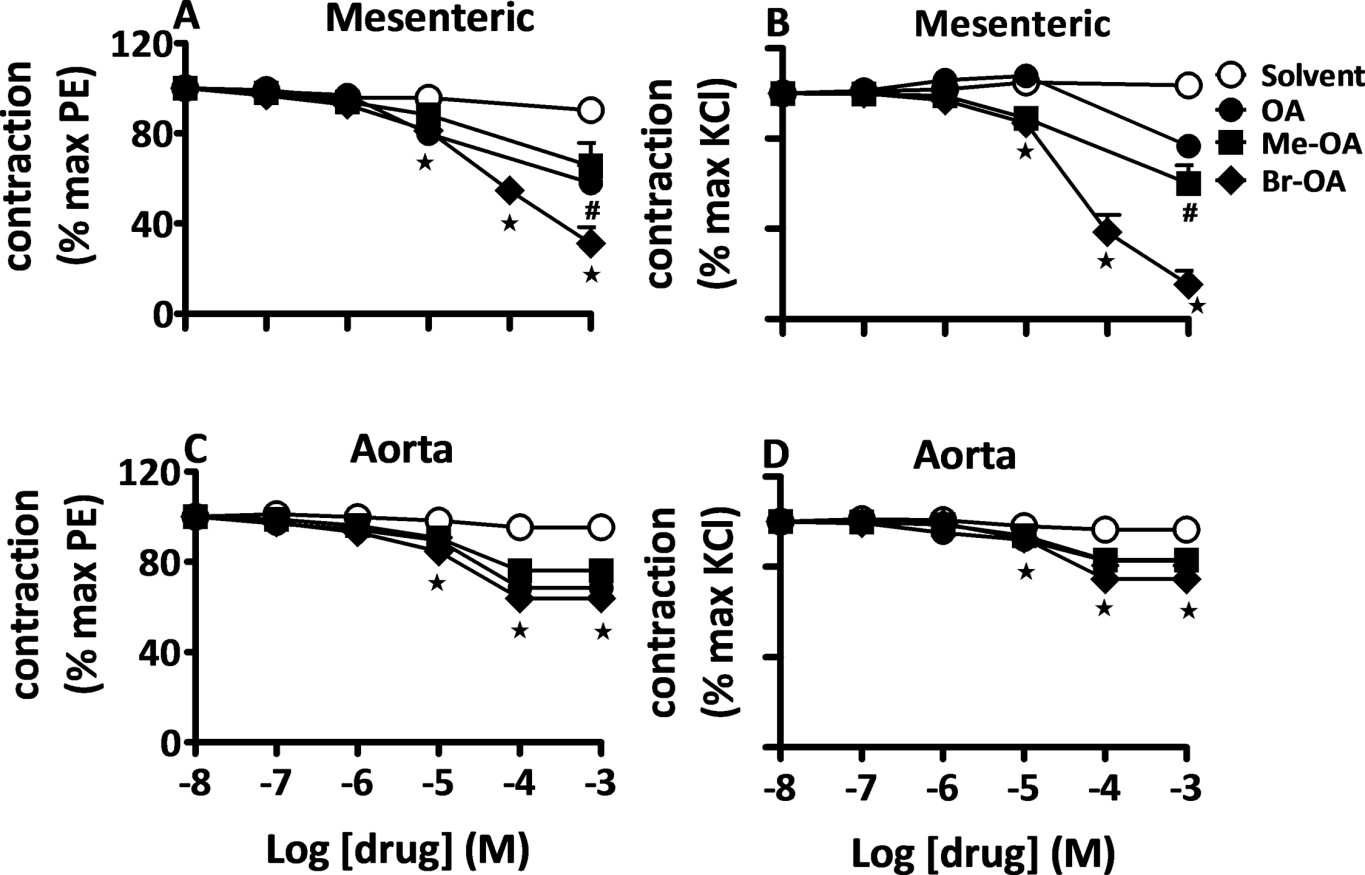
OA and derivatives evoke a vasodilation in aorta and mesenteric arteries from DSS rats. Concentration-response curves for solvent, OA, Me-OA, and Br-OA in endothelium-intact mesenteric arteries [A, B] or aortic rings [C, D] isolated from DSS rats, pre-contracted with sub-maximal concentration of PE (5 μM) [A, C] and KCl (50 mM) [B, D]. The values shown are means ± SEM (n = 7). * p ˂ 0.001 vs control, **#** vs Br-OA.

A similar pattern in relaxation with all the three drugs was observed in arterial segments isolated from DSS rats (Fig 3). There was no difference in the magnitude of relaxant effects of OA and derivatives in Wistar and DSS isolated vessels (e.g. % residual contraction of PE-precontraction of mesenteric arteries 22.03 ±3.43% in Wistar vs. 31.13 ±7.33% in DSS and aortic rings 62.83 ±7.26% in Wistar vs. 63.89 ±4.88% in DSS with 10^−3^ M Br-OA; n = 7; p > 0.05). Further experiments to evaluate the mechanisms of vasorelaxation properties of OA and derivatives were conducted in arterial segments isolated from Wistar rats only.

#### Endothelium-dependent vasorelaxant effects of OA and derivatives

**Fig 4:**
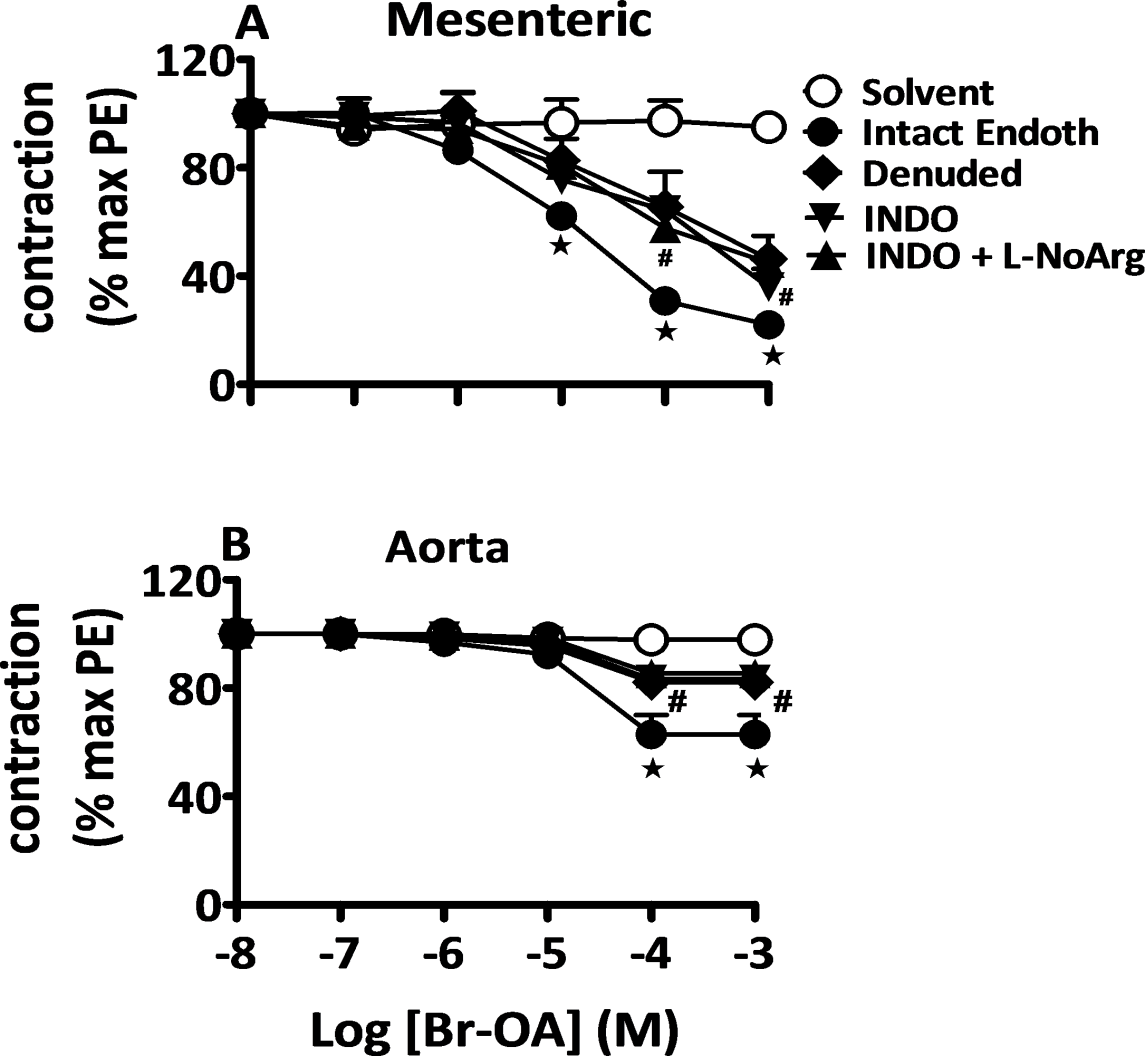
Role of the endothelium in response to Br-OA. Concentration-response curves for solvent and Br-OA in mesenteric arteries (A) and aortic rings (B) isolated from Wistar rats pre-contracted with PE (5 μM). Curves were obtained in endothelium-denuded and intact arteries. Some endothelium-intact vessels were incubated in the presence of INDO (10 μM) only or in combination with L-NoArg (100 μM) prior to addition of the drug. Values shown are means ± SEM (n = 7). * p ˂ 0.001 vs control, **#** vs intact endothelium.

The relaxation evoked by OA and derivatives was only partially inhibited in the absence of functional endothelium as shown in endothelium-denuded vascular rings experiments (Fig 4, shown only for Br-OA, p < 0.05 for mesenteric vessels; p < 0.001 for aorta; results for OA and Me-OA presented in S1 and S2 Figs respectively). This indicates that the relaxation-evoked by OA or derivatives involves both endothelium-dependent and independent mechanisms.

In order to characterize the endothelium-dependent relaxation, endothelium-intact vessels were incubated in the presence of the COX inhibitor INDO or of the NOS inhibitor L-NoArg. As shown in Fig 4, incubation with INDO blocked Br-OA-evoked relaxation to the same extent as removal of the endothelium (p < 0.05 for absence vs. presence of INDO in mesenteric arteries; p < 0.001 for aorta). Addition of L-NoArg did not further inhibit the COX-resistant relaxation. Qualitatively similar results were observed with OA and Me-OA (see S1 and S2 Figs). In a limited number of experiments, vessels were exposed to L-NoArg in the absence of INDO (see S1 Table) without any significant inhibition of the relaxation to OA or derivatives. These data are in favor of an involvement of COX and prostanoids-dependent mechanisms, and against the implication of NOS/NO-dependent pathways.

#### Endothelium-independent vasorelaxant effects of OA and derivatives

**Fig 5:**
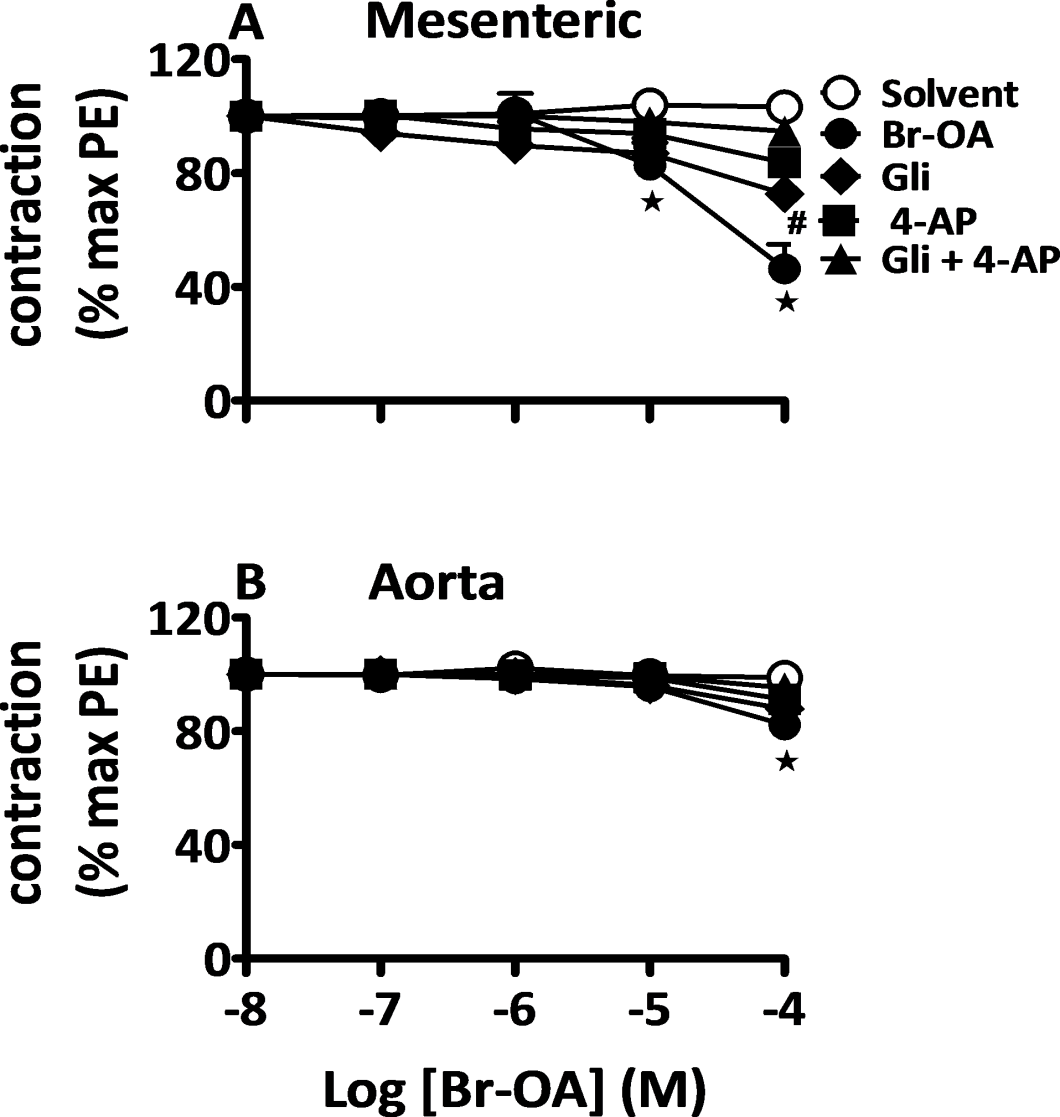
Role of K^+^ channels in response to Br-OA. Concentration-response curves for solvent and Br-OA in denuded mesenteric arteries (A) and aortic rings (B) isolated from Wistar rats pre-contracted with PE (5 μM). Denuded arteries were incubated in the presence of Gli (5 mM), 4-AP (1 mM) or combination of the two inhibitors prior to the addition of the drug. Values shown are means ± SEM (n = 7). * p ˂ 0.001 vs control, **#** vs Br-OA.

**Fig 6:**
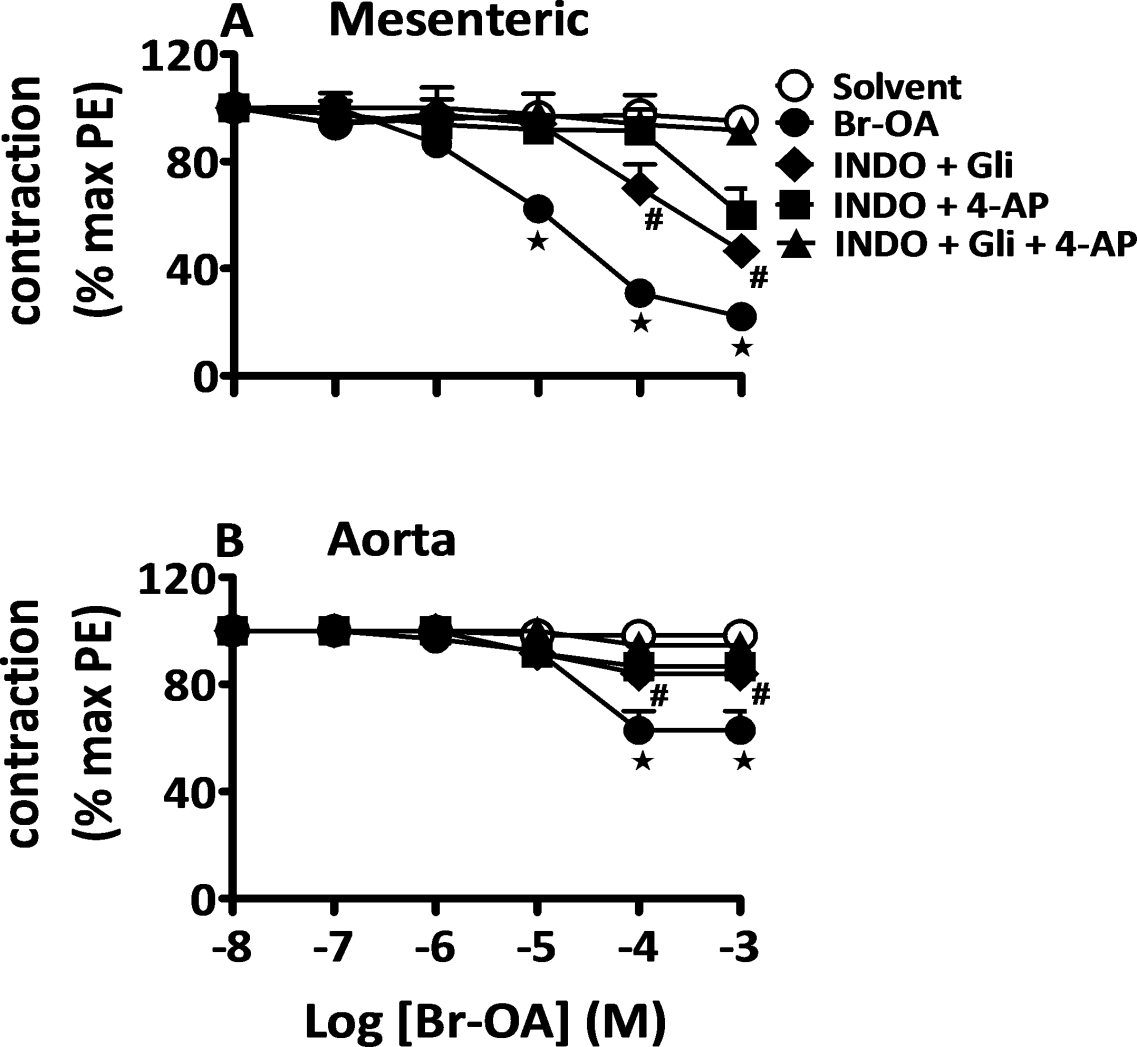
Implication of prostanoids and K^+^ channels in response to Br-OA. Concentration-response curves for solvent and Br-OA in intact mesenteric arteries (A) and aortic rings (B) isolated from Wistar rats pre-contracted with PE (5 μM). Curves in intact arteries incubated in the presence of INDO (10 μM) and Gli (5 mM) or 4-AP (1 mM) and combination of the three inhibitors prior to the addition of the drug. Values shown are means ± SEM (n = 7). * p ˂ 0.001 vs control, **#** vs Br-OA.

As observed above (Fig 4), a significant portion of the relaxation evoked by OA and derivatives is resistant to removal of the endothelium suggesting a role of endothelium-independent pathways. To investigate the possible involvement of K^+^ channels, denuded vessels were incubated in the presence of glibenclamide (Gli) to block ATP-dependent K^+^ channels, and of 4-aminopyridine (4-AP) to block voltage-activated K^+^ channels. Fig 5 shows that either inhibitor when applied alone was able to block part of the relaxation evoked by Br-OA. The effects of both inhibitors were additive since concomitant application of both blockers allowed a complete inhibition of the relaxation caused by Br-OA. The endothelium-independent relaxation evoked by OA and Me-OA was also completely blunted by Gli and 4-AP (see S3 and S4 Figs respectively). Experiments performed with endothelium-intact vessels incubated with a cocktail of INDO, 4-AP and Gli did not show any relaxation upon addition of Br-OA as shown in Fig 6 (illustrated for OA and Me-OA in S5 and S6 Figs respectively). These data suggest that ATP-dependent and voltage-activated K^+^ channels opening mediate the endothelium independent relaxation.

## Discussion

The present study was carried out to further investigate the mechanisms of blood pressure lowering effects of OA and related synthetic derivatives. Previous studies have documented a marked natriuretic action of these drugs in vivo [7, 8, 12]. The present study shows that OA and synthetic derivatives do not decrease but rather increase myocyte shortening in cells isolated from normotensive rats but not in cells isolated from hypertensive animals. The study also confirms previous observations that OA possesses vasorelaxant properties in the aorta and in mesenteric arteries [13, 14], and extend these properties to the two synthetic derivatives, Br-OA and Me-OA.

Cardiac function is one of the factors that regulate mean arterial pressure (MAP), and an effect on the heart, such as a decrease of the heart rate or of the stroke volume, could also contribute to the blood pressure lowering effect of OA and its derivatives. No or mild effects on heart rate have been reported for the compounds in previous studies [15, 16], indicating that bradycardia cannot account for the marked decrease in MAP obtained with the drugs. In the absence of a change in heart rate, a decrease of cardiac output and MAP can be brought about by a change in stroke volume. Such a decrease of stroke volume is unlikely because OA and derivatives did not cause any negative inotropic effect.

In cells isolated from DSS rats the time to peak and relaxation time was higher than that of cells from normotensive rats. Cardiac hypertrophy and heart failure caused by high blood pressure reduce the ability of Ca^2+^ to trigger calcium release from the sarcoplasmic reticulum [17]. This, in addition to the decreased sarcoplasmic reticulum Ca^2+^ uptake [18] may explain the delayed time-to-peak and relaxation in cardiomyocytes from DSS rats. OA and derivatives had no apparent effect on shortening in these animals, indicating that the mechanism underlying the OA and derivatives-induced positive inotropic effect in Wistar rats is deficient in DSS animals. The mechanism underlying this positive inotropic effect was not clarified. We did not observe any increase of L-type Ca^2+^ currents. The experiments on I_Ca_ were carried out at room temperature, using cardiomyocytes dialyzed under whole-cell patch-clamp conditions. Additional studies using cells with preserved intracellular medium (under perforated patch conditions) and at 37°C are needed before completely excluding a mechanism involving L-type Ca^2+^ currents. In any case, our study suggests that a negative inotropic action, resulting in a decrease of stroke volume, is excluded as a mechanism for the hypotensive action of OA and derivatives. On the other hand, increased cardiomyocyte contractility implies an increase in cardiac output, an effect which may be beneficial for a drug which causes natriuresis and vasodilation.

The relaxation evoked by OA and derivatives was only partially inhibited in endothelium-denuded vascular rings. This demonstrates that in our experimental setting the relaxation involves both endothelium-dependent and independent mechanisms. Other reports suggest that OA-induced relaxation is nearly fully endothelium-dependent and is associated with an increased production of NO since relaxation was marginal in endothelium-denuded vessels and it could be abrogated by NO synthase inhibitors or by NO scavengers [19]. Our results are thus in contradiction with these previous findings, despite the fact that the various studies used the same types of vessels (aorta and mesenteric arteries) from the same species (rat). There is no clear explanation for this discrepancy. However, the same research groups also reported substantial (_~_34%) endothelium-resistant relaxation [14, 20]. One possible reason for the difference might be due to the fact that in our study we tested the relaxation in PE-precontracted vessels. PE was also the agonist used to contract the vessels in the above-mentioned studies which also reported substantial endothelium-resistant relaxation [14, 20]. This is in contrast with the studies where the vessels were precontracted with norepinephrine [19]. Previous studies have noted that vascular effects of norepinephrine may not only implicate the effects on alpha-adrenergic receptors, but also effects due to beta-adrenergic receptors and in addition effects due to non-adrenergic (e.g. dopaminergic) receptors. Therefore, despite the fact that the use of norepinephrine is a better mimic of the physiological conditions, phenylephrine might be more appropriate when deciphering the mechanisms involved in drug action [21]. Noteworthy, our results demonstrate that the endothelium-dependent fraction of the relaxation to OA and derivatives is abrogated in the presence of COX inhibitor attesting the implication of a prostanoid-dependent pathway. Our results corroborate previous studies [22] suggesting that OA promotes prostaglandin I_2_ release from vascular smooth muscle cells albeit in a different time-scale.

Pathways underlying the endothelium-independent relaxation evoked by OA and derivatives may implicate the opening of the K^+^ channels. Therefore, we tested this hypothesis in denuded vessels incubated in the presence of blockers of different K^+^ channels types (Gli for ATP-dependent K^+^ channels and 4-AP for voltage-activated K^+^ channels). Either inhibitor when applied alone blunted part of the relaxation evoked by OA and derivatives. The effects of both inhibitors were additive and their simultaneous application allowed a complete blockade of the relaxation to OA, Br-OA and Me-OA, indicating that ATP-dependent and voltage-activated K^+^ channel opening in vascular smooth muscle mediate the endothelium independent relaxation.

The present study tested whether two synthetic derivatives of OA display effects similar to those of the parent compound. Interestingly, the maximum relaxation evoked by one of the derivatives, Br-OA, was significantly larger in the mesenteric vessels although these differences were not observed in the aorta. The difference seems to be largely due to the endothelium-independent component but the mechanism underlying the larger efficacy of Br-OA is not known. We speculate that this variation in relaxation profiles of these compounds is probably due to differences in the effects on receptors or effectors (e.g. channels) present.

The OA or derivatives-induced vasodilatation is expected to result in the decrease of total peripheral resistance which could be implicated in the blood pressure lowering effect of these triterpenes. However, in vivo studies indicate that OA, Br-OA or Me-OA acutely decrease blood pressure to a similar extent [8]. A similar hypotensive effect is not expected if Br-OA is more efficacious at inducing the vasodilatatory action as suggested by the present study. Additional factors may contribute to or interfere with the vasodilatatory action. We speculate that the previously reported improved Na^+^ elimination caused by these compounds could interact with these effects on vascular function. Natriuresis without increased diuresis as was obtained in our in vivo studies [8] is expected to induce a decrease in extracellular Na^+^ concentration which can affect transmembrane Na^+^ gradients and influence the activity of the Na^+^-Ca^2+^ exchanger, resulting in changed Ca^2+^ movements via this pathway. The resulting changes in intracellularly available Ca^2+^ could influence relaxation and hence affect the other mechanisms that decrease vascular resistance.

In conclusion, the present study provides evidence that OA, Me-OA and Br-OA possess vasodilatory actions mediated in part by the endothelium-dependent COX/prostanoids pathway, but also in part by endothelium-independent opening of ATP-dependent and voltage-activated K^+^ channels. It sheds further light on possible mechanisms for the antihypertensive effects of OA and related synthetic oleanane derivatives, which may involve a decrease in vascular resistance.

## Supporting Information

S1 Fig Role of the endothelium in the response to OA. Concentration-response curves for solvent and OA in mesenteric arteries (A) and aortic rings (B) isolated from Wistar rats pre-contracted with PE (5 μM). Curves were obtained in endothelium-denuded and intact arteries. Some endothelium-intact vessels were incubated in the presence of INDO (10 μM) only or in combination with L-NoArg (100 μM) prior to addition of the drug. Values shown are means ± SEM (n = 7). * p ˂ 0.001 vs control, **#** p ˂ 0.001 vs cells from normotensive Wistar rats.
(TIF)

S2 Fig Role of the endothelium in the response to Me-OA. Concentration-response curves for solvent and Me-OA in mesenteric arteries (A) and aortic rings (B) isolated from Wistar rats pre-contracted with PE (5 μM). Curves were obtained in endothelium-denuded and intact arteries. Some endothelium-intact vessels were incubated in the presence of INDO (10 μM) only or in combination with L-NoArg (100 μM) prior to addition of the drug. Values shown are means ± SEM (n = 7). * p ˂ 0.001 vs control.
(TIF)

S3 Fig Role of K^+^ channels in the response to OA. Concentration-response curves for solvent and OA in denuded mesenteric arteries (A) and aortic rings (B) isolated from Wistar rats pre-contracted with PE (5 μM). Denuded arteries were incubated in the presence of Gli (5 mM), 4-AP (1 mM) or combination of the two inhibitors prior to the addition of the drug. Values shown are means ± SEM (n = 7). * p ˂ 0.001 vs control.
(TIF)

S4 Fig Role of K^+^ channels in the response to Me-OA. Concentration-response curves for solvent and Me-OA in denuded mesenteric arteries (A) and aortic rings (B) isolated from Wistar rats pre-contracted with PE (5 μM). Denuded arteries were incubated in the presence of Gli (5 mM), 4-AP (1 mM) or combination of the two inhibitors prior to the addition of the drug. Values shown are means ± SEM (n = 7). * p ˂ 0.001 vs control.
(TIF)

S5 Fig Implication of prostanoids and K^+^ channels in the response to OA. Concentration-response curves for solvent and OA in intact mesenteric arteries (A) and aortic rings (B) isolated from Wistar rats pre-contracted with PE (5 μM). Curves in intact arteries incubated in the presence of INDO (10 μM) and Gli (5 mM) or 4-AP (1 mM) and combination of the three inhibitors prior to the addition of the drug. Values shown are means ± SEM (n = 7). * p ˂ 0.001 vs control.
(TIF)

S6 Fig Implication of prostanoids and K^+^ channels in the response to Me-OA. Concentration-response curves for solvent and Me-OA in intact mesenteric arteries (A) and aortic rings (B) isolated from Wistar rats pre-contracted with PE (5 μM). Curves in intact arteries incubated in the presence of INDO (10 μM) and Gli (5 mM) or 4-AP (1 mM) and combination of the three inhibitors prior to the addition of the drug. Values shown are means ± SEM (n = 7). * p ˂ 0.001 vs control.
(TIF)

S1 Table Role of endothelium in response to OA, Me-OA and Br-OA. Preliminary concentration-responses for OA, Me-OA, and Br-OA (30 μM) in endothelium-intact mesenteric arteries or aortic rings isolated from Wistar rats, pre-contracted with sub-maximal concentration of PE (5 μM). Vessels were incubated in the presence of L-NoArg (100 μM) prior to addition of the drug. The values shown are means ± SEM (n = 6).
(TIF)

## References

[1] Drummond GR, Selemidis S, Griendling KK, Sobey CG. Combating oxidative stress in vascular disease: NADPH oxidases as therapeutic targets. Nature Reviews Drug Discovery 2011; 10(6): 453–471. 10.1038/nrd3403 21629295 PMC3361719

[2] Mathers CD, Loncar D. Projections of global mortality and burden of disease from 2002 to 2030. PLoS Medicine 2006; 3(11): e442–e462. 17132052 10.1371/journal.pmed.0030442 PMC1664601

[3] Gao D, Li Q, Li Y, Liu Z, Fan Y, Liu Z, et al Antidiabetic and antioxidant effects of oleanolic acid from *Ligustrum lucidum* Ait in alloxan-induced diabetic rats. Phytotherapy Research 2009; 23: 1257–1262. 10.1002/ptr.2603 19274687

[4] Kamboj VP. Herbal medicine. Current Science 2000; 78(1): 35–51.

[5] Ozsoy-Sacan O, Karabulut-Bulan O, Bolkent S, Yanardag R, Ozgey Y. Effects of chard (*Beta vulgaris* L. var cicla) on the liver of the diabetic rats: A morphological and biochemical study. Bioscience, Biotechnology and Biochemistry 2004; 68: 1640–1648. 10.1271/bbb.68.1640 15322346

[6] Martín R, Hernández M, Córdova C, Nieto ML. Natural triterpenes modulate immune‐inflammatory markers of experimental autoimmune encephalomyelitis: Therapeutic implications for multiple sclerosis. British Journal of Pharmacology 2012; 166(5): 1708–1723. 10.1111/j.1476-5381.2012.01869.x 22260389 PMC3419913

[7] Madlala HP, Masola B, Singh M, Musabayane CT. The effects of *Syzygium aromaticum*-derived oleanolic acid on kidney function of male Sprague-Dawley rats and on kidney and liver cell lines. Renal Failure 2012; 34: 767–776. 10.3109/0886022X.2012.678172 22512664

[8] Madlala HP, Van Heerden FR, Mubagwa K, Musabayane CT. Changes in renal function and oxidative status associated with the hypotensive effects of oleanolic acid and related synthetic derivatives in experimental animals. PloS One 2015; 10(6): 1–20. 10.1371/journal.pone.0128192 PMC4457832 26046776

[9] Gwanyanya A, Sipido KR, Vereecke J, Mubagwa K. ATP and PIP2 dependence of the magnesium-inhibited, TRPM7-like cation channel in cardiac myocytes. American Journal of Cell Physiology 2006; 291: C627–C635. 10.1152/ajpcell.00074.2006 16707555

[10] Martinsen A, Baeyens N, Yerna X, Morel N. Rho kinase regulation of vasopressin-induced calcium entry in vascular smooth muscle cell: Comparison between rat isolated aorta and cultured aortic cells. Cell Calcium 2012; 52(6): 413–421. 10.1016/j.ceca.2012.07.002 22883550

[11] Mulvany MJ, Halpern W. Contractile properties of small arterial resistance vessels in spontaneously hypertensive and normotensive rats. Circulation Research 1977; 41(1): 19–26. 862138 10.1161/01.res.41.1.19

[12] Mapanga RF, Tufts MA, Shode FO, Musabayane CT. Renal effects of plant‐derived oleanolic acid in streptozotocin-induced diabetic rats. Renal Failure 2009; 31: 481–491. 19839826 10.1080/08860220902963558

[13] Dongmoa AB, Azebazeb AGB, Donfackc FM, Dimoc T, Nkeng-Efouetd PA, Devkotae KP, et al Pentacyclic triterpenoids and ceramide mediate the vasorelaxant activity of *Vitex cienkowskii* via involvement of NO/cGMP pathway in isolated rat aortic rings. Journal of Ethnopharmacology 2011; 133: 204–212. 10.1016/j.jep.2010.09.033 20920567

[14] Rodriguez-Rodriguez R, Perona JS, Herrera MD, Ruiz-Gutierrez V. Triterpenic compounds from “orujo” olive oil elicit vasorelaxation in aorta from spontaneously hypertensive rats. Journal of Agricultural and Food Chemistry 2006; 54(6): 2096–2102. 16536581 10.1021/jf0528512

[15] Somova LO, Nadar A, Rammanan P, Shode FO. Cardiovascular, antihyperlipidemic and antioxidant effects of oleanolic and ursolic acids in experimental hypertension. Phytomedicine 2003; 10: 115–121. 12725563 10.1078/094471103321659807

[16] Somova L, Shode F, Mipando M. Cardiotonic and antidysrhythmic effects of oleanolic and ursolic acids, methyl maslinate and uvaol. Phytomedicine 2004; 11(2): 121–129. 15070161 10.1078/0944-7113-00329

[17] Gomez AM, Valdivia HH, Cheng H, Lederer MR, Santana LF, Cannell MB, et al Defective excitation-contraction coupling in experimental cardiac hypertrophy and heart failure. Science 1997; 276(5313): 800–806. 9115206 10.1126/science.276.5313.800

[18] Houser SR, Piacentino V III, Weisser J. Abnormalities of calcium cycling in the hypertrophied and failing heart. Journal of molecular and cellular cardiology 2000; 32(9): 1595–1607. 10966823 10.1006/jmcc.2000.1206

[19] Rodriguez‐Rodriguez R, Stankevicius E, Herrera M, Ostergaard L, Andersen MR, Ruiz‐Gutierrez V, et al Oleanolic acid induces relaxation and calcium‐independent release of endothelium‐derived nitric oxide. British Journal of Pharmacology 2008; 155(4): 535–546. 10.1038/bjp.2008.289 18622409 PMC2579672

[20] Rodríguez-Rodríguez R, Herrera MD, Perona JS, Ruiz-Gutiérrez V. Potential vasorelaxant effects of oleanolic acid and erythrodiol, two triterpenoids contained in ‘orujo’olive oil, on rat aorta. British Journal of Nutrition 2004; 92(04): 635–642. 15522132 10.1079/bjn20041231

[21] Van der Graaf PH, Saxena PR, Shankley NP, Black JW. Exposure and characterization of the action of noradrenaline at dopamine receptors mediating endothelium‐independent relaxation of rat isolated small mesenteric arteries. British Journal of Pharmacology 1995; 116(8): 3237–3242. 8719802 10.1111/j.1476-5381.1995.tb15130.x PMC1909169

[22] Martinez-Gonzalez J, Rodriguez-Rodriguez R, Gonzalez-Diez M, Rodriguez C, Herrera MD, Ruiz-Gutierrez V, et al Oleanolic acid induces prostacyclin release in human vascular smooth muscle cells through a cyclooxygenase-2-dependent mechanism. The Journal of Nutrition 2008; 138(3): 443–448. 18287347 10.1093/jn/138.3.443

