# Supplementary figures and images for: Vascular Endothelium-Dependent and Independent Actions of Oleanolic Acid and Its Synthetic Oleanane Derivatives as Possible Mechanisms for Hypotensive Effects
Source: PLoS One. 2016 Jan 22;11(1):e0147395. doi: 10.1371/journal.pone.0147395 (PMC4723044; doi:10.1371/journal.pone.0147395)

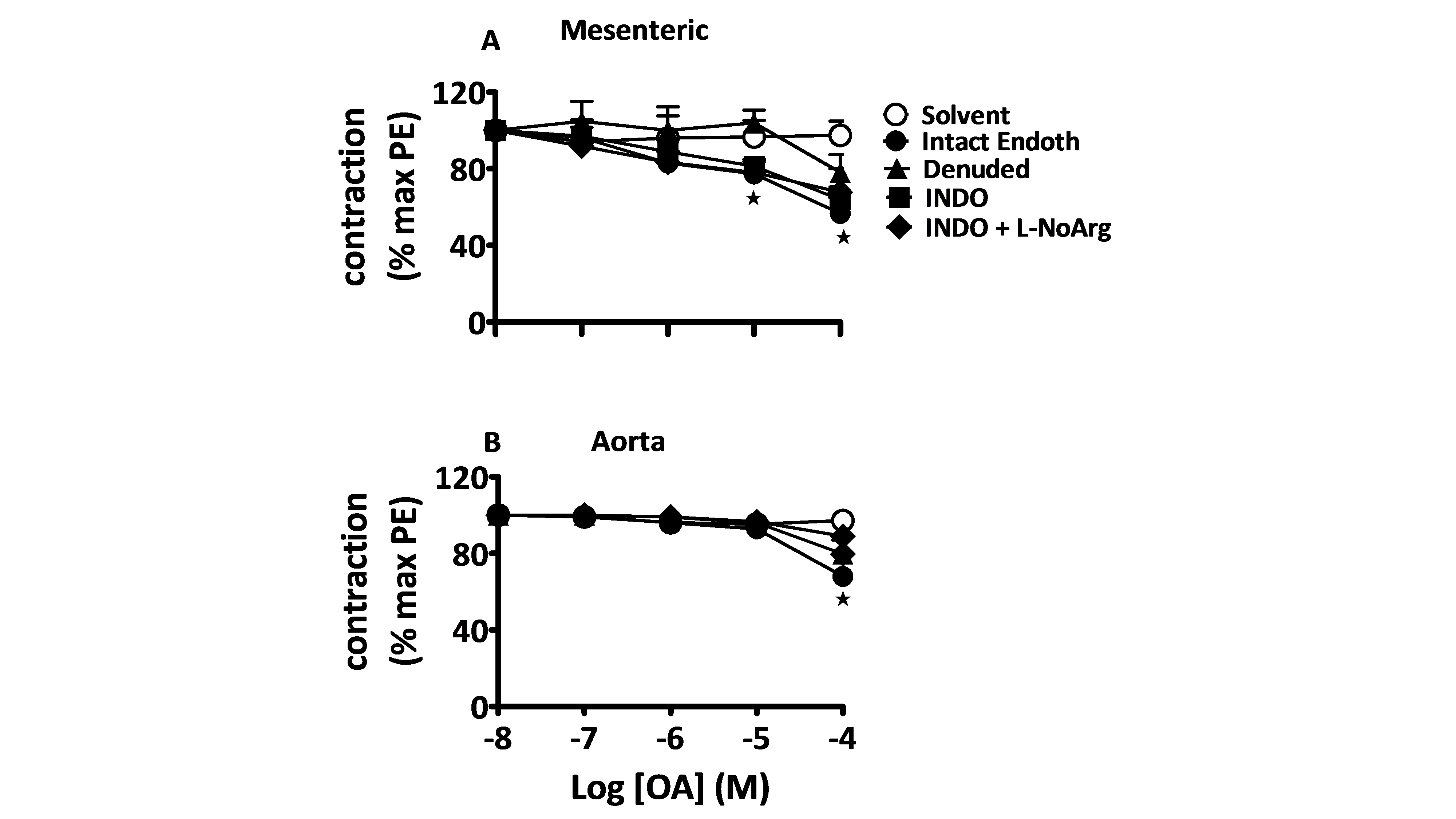

Supplement: S1 Fig — Concentration-response curves for solvent and OA in mesenteric arteries (A) and aortic rings (B) isolated from Wistar rats pre-contracted with PE (5 μM). Curves were obtained in endothelium-denuded and intact arteries. Some endothelium-intact vessels were incubated in the presence of INDO (10 μM) only or in combination with L-NoArg (100 μM) prior to addition of the drug. Values shown are means ± SEM (n = 7). * p ˂ 0.001 vs control, # p ˂ 0.001 vs cells from normotensive Wistar rats. (TIF) [file pone.0147395.s001.tif]

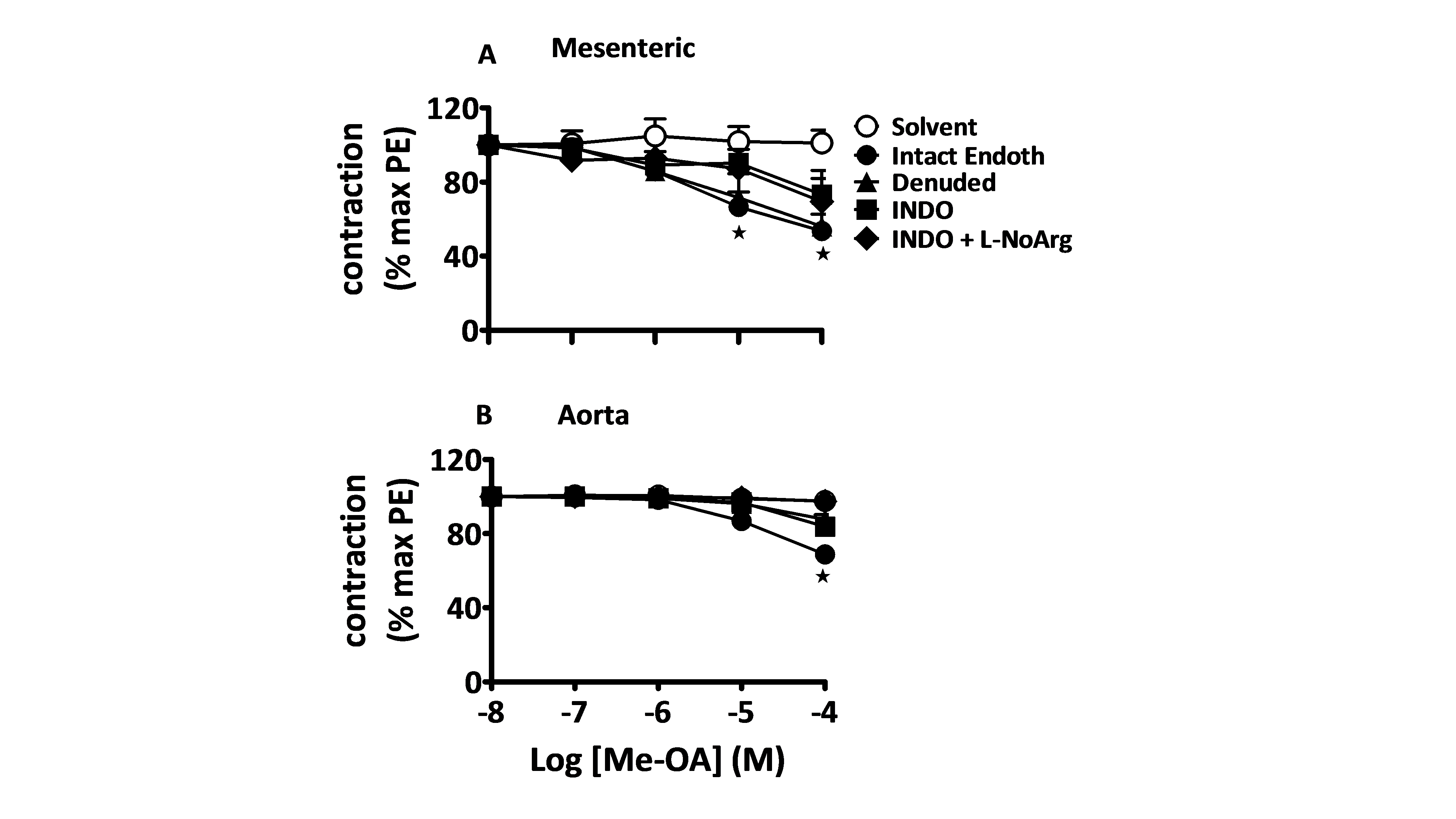

Supplement: S2 Fig — Concentration-response curves for solvent and Me-OA in mesenteric arteries (A) and aortic rings (B) isolated from Wistar rats pre-contracted with PE (5 μM). Curves were obtained in endothelium-denuded and intact arteries. Some endothelium-intact vessels were incubated in the presence of INDO (10 μM) only or in combination with L-NoArg (100 μM) prior to addition of the drug. Values shown are means ± SEM (n = 7). * p ˂ 0.001 vs control. (TIF) [file pone.0147395.s002.tif]

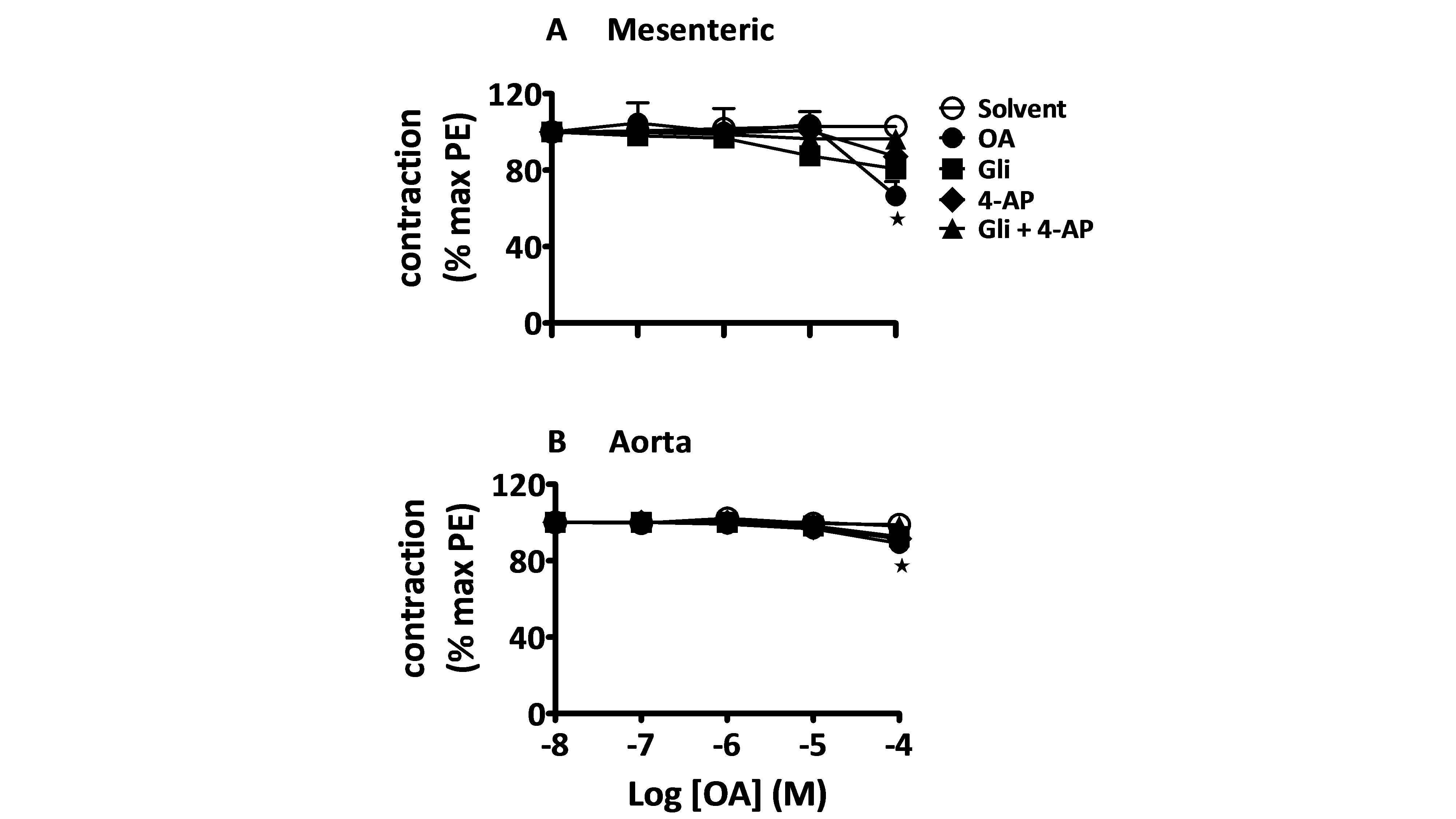

Supplement: S3 Fig — Concentration-response curves for solvent and OA in denuded mesenteric arteries (A) and aortic rings (B) isolated from Wistar rats pre-contracted with PE (5 μM). Denuded arteries were incubated in the presence of Gli (5 mM), 4-AP (1 mM) or combination of the two inhibitors prior to the addition of the drug. Values shown are means ± SEM (n = 7). * p ˂ 0.001 vs control. (TIF) [file pone.0147395.s003.tif]

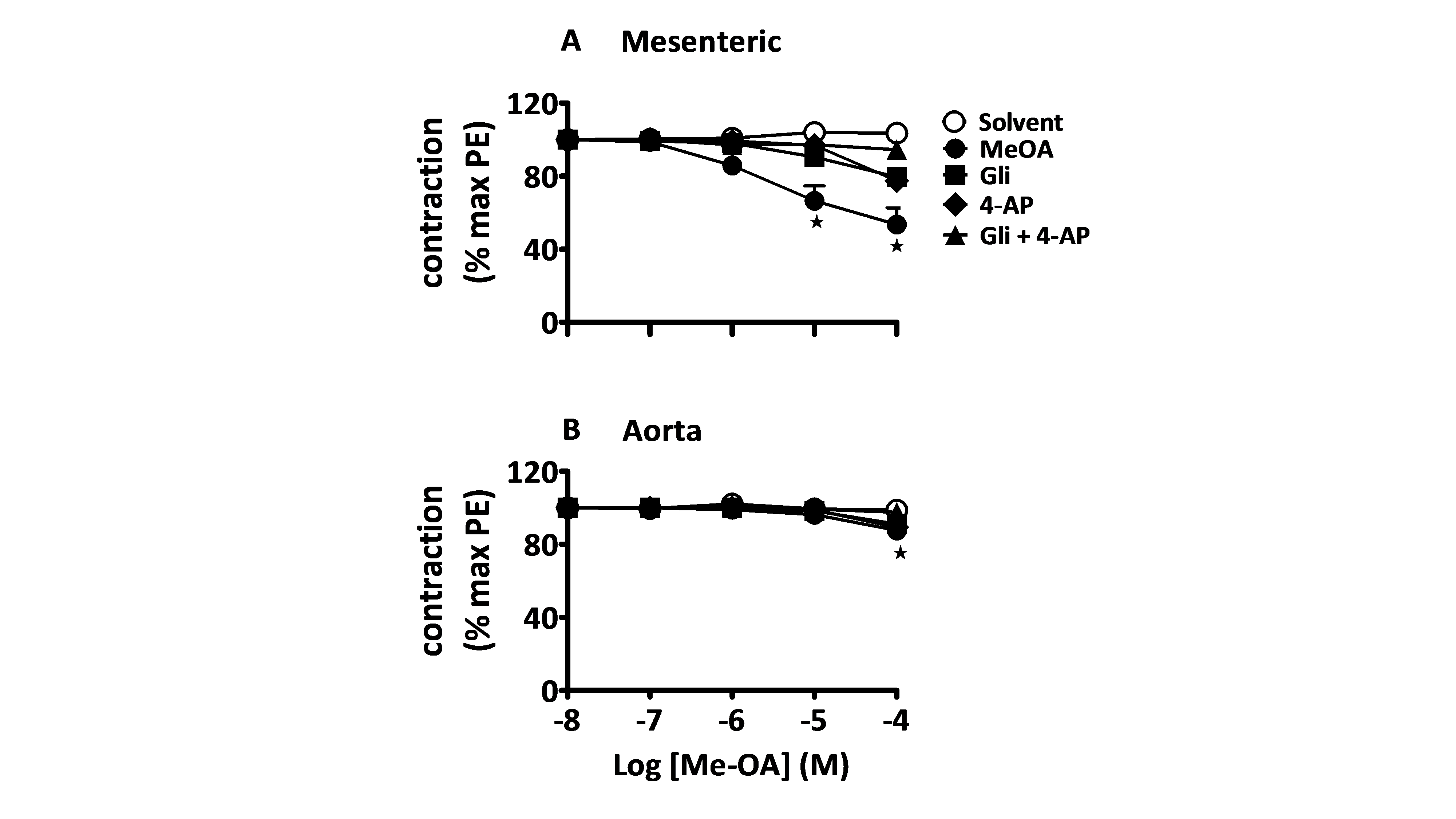

Supplement: S4 Fig — Concentration-response curves for solvent and Me-OA in denuded mesenteric arteries (A) and aortic rings (B) isolated from Wistar rats pre-contracted with PE (5 μM). Denuded arteries were incubated in the presence of Gli (5 mM), 4-AP (1 mM) or combination of the two inhibitors prior to the addition of the drug. Values shown are means ± SEM (n = 7). * p ˂ 0.001 vs control. (TIF) [file pone.0147395.s004.tif]

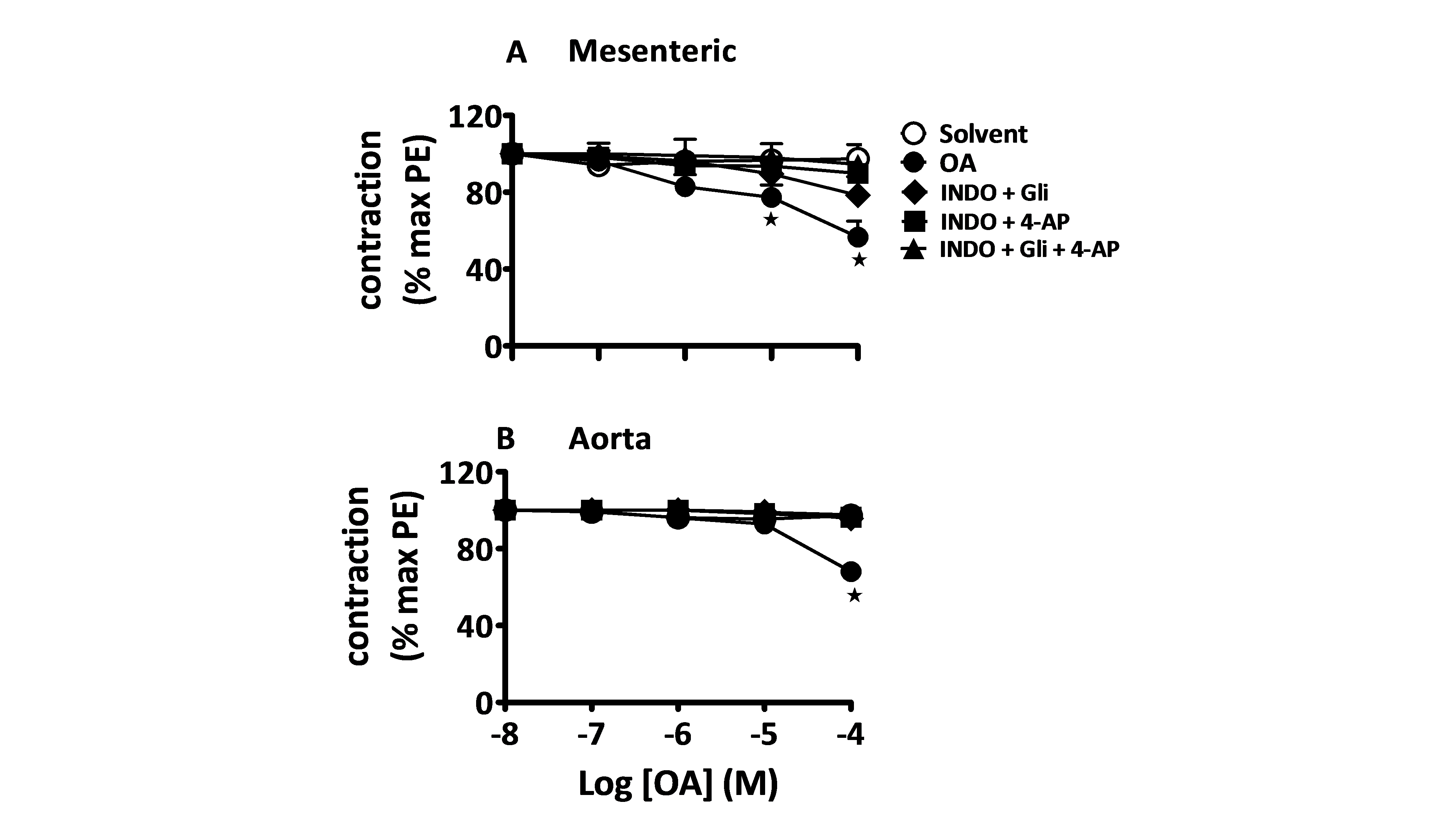

Supplement: S5 Fig — Concentration-response curves for solvent and OA in intact mesenteric arteries (A) and aortic rings (B) isolated from Wistar rats pre-contracted with PE (5 μM). Curves in intact arteries incubated in the presence of INDO (10 μM) and Gli (5 mM) or 4-AP (1 mM) and combination of the three inhibitors prior to the addition of the drug. Values shown are means ± SEM (n = 7). * p ˂ 0.001 vs control. (TIF) [file pone.0147395.s005.tif]

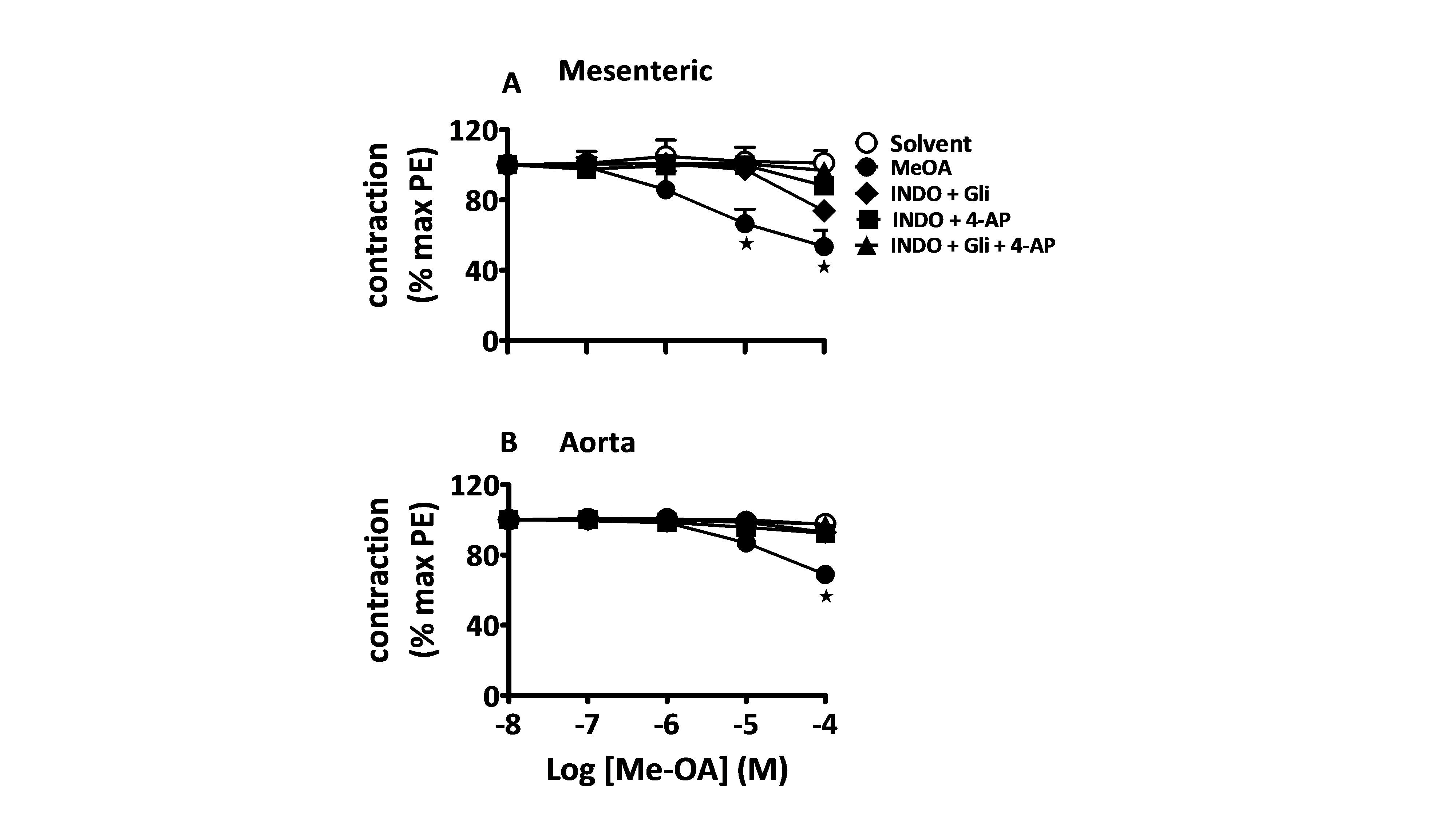

Supplement: S6 Fig — Concentration-response curves for solvent and Me-OA in intact mesenteric arteries (A) and aortic rings (B) isolated from Wistar rats pre-contracted with PE (5 μM). Curves in intact arteries incubated in the presence of INDO (10 μM) and Gli (5 mM) or 4-AP (1 mM) and combination of the three inhibitors prior to the addition of the drug. Values shown are means ± SEM (n = 7). * p ˂ 0.001 vs control. (TIF) [file pone.0147395.s006.tif]

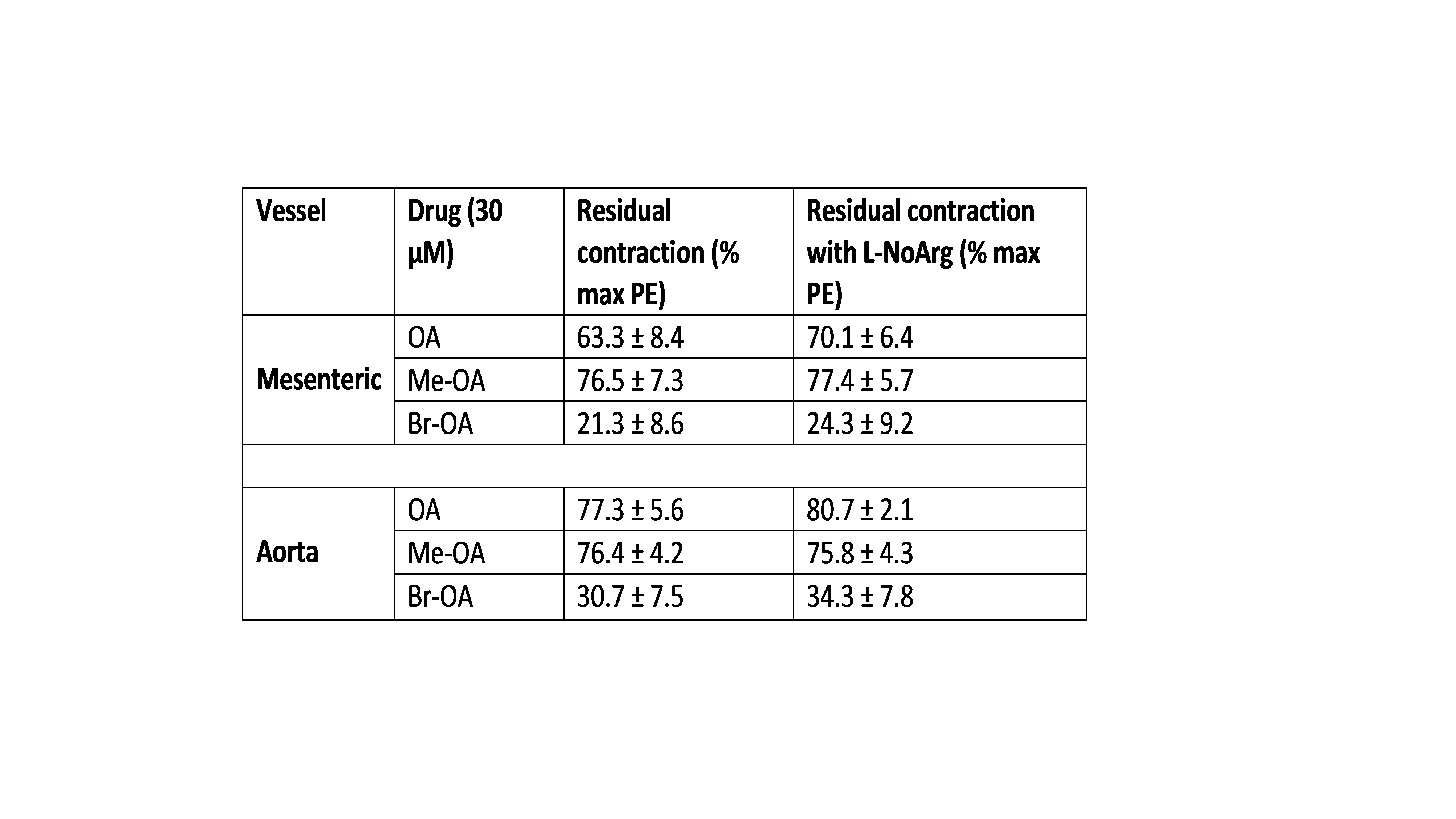

Supplement: S1 Table — Preliminary concentration-responses for OA, Me-OA, and Br-OA (30 μM) in endothelium-intact mesenteric arteries or aortic rings isolated from Wistar rats, pre-contracted with sub-maximal concentration of PE (5 μM). Vessels were incubated in the presence of L-NoArg (100 μM) prior to addition of the drug. The values shown are means ± SEM (n = 6). (TIF) [file pone.0147395.s007.tif]
